# Immunoglobulin-induced aseptic meningitis: a case report

**DOI:** 10.1186/s12883-018-1102-8

**Published:** 2018-07-17

**Authors:** Luísa Graça, Joana Alves, João Nuak, António Sarmento

**Affiliations:** 1 Infectious Diseases Department, Centro Hospitalar de São João, Alameda Prof. Hernâni Monteiro, 4202-451 Porto, Portugal; 2 Instituto Nacional de Engenharia Biomédica (INEB), Instituto de Inovação e Investigação em Saúde (I3S), Grupo de I&D em Nefrologia e Doenças Infecciosas, Porto, Portugal; 3 Department of Medicine, Faculty of Medicine, University of Porto, Porto, Portugal

**Keywords:** Aseptic meningitis, Hypogammaglobulinemia, Intravenous immunoglobulin, Systemic lupus erythematous

## Abstract

**Background:**

Immunoglobulin associated meningitis is a rare disease that mimics infectious meningitis. This is, to our knowledge, the first case of Immunoglobulin-associated meningitis described in a patient with Systemic Lupus Erythematosus and hypogammaglobulinemia secondary to Rituximab.

**Case presentation:**

A 46-year-old female with a past medical history of Systemic Lupus Erythematosus, presented with meningismus 36 h after first infusion of intravenous immunoglobulin. The cerebrospinal fluid analysis showed neutrophilic pleocytosis and hyperproteinorrachia. All microbiological tests were negative. The patient recovered remarkably fast without sequela after just five days of antibiotic therapy.

**Conclusion:**

Systemic Lupus Erythematosus is a well-documented risk factor for aseptic meningitis associated with other drugs. Possibly, it is also a risk factor for Immunoglobulin associated meningitis. This diagnosis, although rare, should be considered in patients receiving Immunoglobulin since it is a self-limited condition and treatment is supportive.

## Background

Drug associated aseptic meningitis is a rare complication of many drugs. It usually resembles infectious meningitis, therefore presenting a diagnostic challenge [1]. Establishing a causal relationship between the use of a drug and the onset of symptoms is the hallmark of diagnosis and it is supported by both negative tests for infectious causes and quick resolution of symptoms after drug discontinuation [2].

The medicines most commonly associated with aseptic meningitis are non-steroidal anti-inflammatory drugs (NSAIDs), antibiotics, intravenous immunoglobulin (IVIG) and OKT3 antibodies [3]. The frequency of IVIG associated meningitis varies widely, from 0 to 11% with a recent study suggesting an overall incidence of 0.60% [1].

Although Moris and colleagues reported a higher incidence of drug associated meningitis in patients with Systemic Lupus Erythematosus (SLE) (29 out of 194 cases), the culprit drug was never IVIG [3, 4]. This is, to our knowledge, the first case of IVIG-associated meningitis described in a patient with SLE and hypogammaglobulinemia secondary to Rituximab.

## Case presentation

A 46-year-old female with a past medical history of SLE and associated Sjögren syndrome, usual interstitial pneumonia and migraine, medicated with prednisolone 5 mg and hydroxicloroquine 400 mg, with a recently worsened asymptomatic hypogammaglobulinemia (IgG of 297 mg/dL and IgA < 8 mg/dL) secondary to rituximab (taken 4 years earlier), was proposed for replacement therapy with IVIG. She had no previous history of therapy with IVIG. She was started on IVIG 10%, 2 g/Kg over 5 consecutive days. She was given two doses of IVIG in two consecutive days without any immediate reaction.

The patient presented to the emergency department, 36 h after the first infusion, with headache, photophobia, nausea, vomiting and fever. On examination, she was prostrated and had neck stiffness without focal neurological signs. Blood work showed low inflammatory parameters. The brain CT was normal. The CSF analysis showed neutrophilic pleocytosis with 1547 cells/mm^3^ (87.5% neutrophils), hyperproteinorrachia (15.3 mg/dL) and mildly reduced glucose (50 mg/dL in CSF and 113 mg/dL in plasma). The patient was admitted to the Infectious Diseases Department with the diagnosis of meningitis and given ceftriaxone 2 g every 12 h and ampicillin 2 g every 4 h.

Blood cultures were negative as well as Gram stain, India ink smear, CSF culture for bacteria and fungus and Nucleic Acid Amplification Test (NAAT) for *Listeria monocytogenes* in the CSF. The urinary pneumococcal antigen was also negative.

The patient was asymptomatic after 2 days of therapy. The lumbar puncture was repeated after 5 days of therapy. The CSF analysis showed 0 cells, normal glucose (67 mg/dL in CSF and 91 mg/dL in plasma) and normal proteins (3.5 mg/dL). Accordingly, the antibiotics were withdrawn and the patient was discharged.

Drug-induced aseptic meningitis usually manifests as meningismus within 48 h after drug exposure. Typically, CSF examination reveals neutrophilic pleocytosis (median 147, range 8–19,000 cells/mm^3^), protein elevation (median 1.20, range 0.04–3.90 g/L) and normal levels of glucose (median 61.64 mg/dL, range 43.45–157.45 mg/dL). Eosinophilic pleocytosis has been reported in some patients. CSF culture is necessarily negative.

In our case, the diagnosis was based on the presence of risk factors for IVIG associated meningitis (migraine, SLE, first infusion and high dose IVIG), the strong temporal relationship between administration of IVIG and onset of symptoms, the typical CSF characteristics, the exclusion of alternative causes and the quick improvement within a few days.

## Discussion and conclusions

This short paper reports, to our knowledge, the first case of immunoglobulin induced aseptic meningitis, a rare complication of IVIG, in a patient with SLE and hypogammaglobulinemia secondary to Rituximab.

The first association of aseptic meningitis with IVIG was reported in 1988, and more than 30 such reports have followed. It has been reported in many indications including Idiopathic Thrombocytopenic Purpura [5], Myasthenia Gravis [6], Inflammatory Demyelinating Neuropathy [7] and Guillain-Barré syndrome [8], but not ever in a patient with SLE and hypogammaglobulinemia secondary to Rituximab.

Patients appear to be at highest risk after the first administration, especially if receiving rapid, high-dose infusion of IVIG [1, 7]. A previous history of migraine seems to be an important predisposing condition [7]. SLE is a well-documented risk factor for aseptic meningitis associated with NSAIDs and other drugs [4]. Although the cause of this association is unknown, SLE may, as well, be a risk factor for IVIG-associated aseptic meningitis. Nevertheless, more studies are needed to confirm this hypothesis.

The pathophysiology of IVIG-associated meningitis is unclear. There are numerous possible mechanisms, including leptomeningeal hypersensitivity reaction, complement direct meningeal irritation triggered by IgG, or interactions between IgG and meningeal vessel antigens causing inflammatory cytokine release [1, 5]. The neurologic symptoms parallel the concentration of IgG in the CSF [9].

IVIG is a rare but clinically relevant cause of aseptic meningitis that is often under recognized. Unlike infectious meningitis, it is self-limited and treatment is supportive.

This case report highlights the importance of this diagnosis in order to avoid unnecessary procedures and therapies.

## Abbreviations

CSF: Cerebrospinal fluid
CT: Computed tomography
IVIG: Intravenous immunoglobulin
NAAT: Nucleic acid amplification test
NSAIDs: Nonsteroidal anti-inflammatory drugs
SLE: Systemic lupus erythematosus
